# Investigating the Mental Health Impacts of University Campus Green Space Through Perceived Sensory Dimensions and the Mediation Effects of Perceived Restorativeness on Restoration Experience

**DOI:** 10.3389/fpubh.2020.578241

**Published:** 2020-12-22

**Authors:** Fahimeh Malekinezhad, Paul Courtney, Hasanuddin bin Lamit, Mauro Vigani

**Affiliations:** ^1^Countryside and Community Research Institute, University of Gloucestershire, Cheltenham, United Kingdom; ^2^Faculty of Built Environment and Surveying, Universiti Teknologi Malaysia, Johor Bahru, Malaysia

**Keywords:** restoration experience, mental health, perceived restorativeness, perceived sensory dimension (PSD), PLS-SEM analysis

## Abstract

**Introduction:** Green spaces support people mentally in their everyday life. Perceived restorativeness and Perceived Sensory Dimension (PSD) have been addressed as optimal environmental related characteristics with regards to psychological restoration. However, relatively little research has investigated how the perception of these characteristics, directly and indirectly, affects restoration experience, particularly in a sample of university students within the area of green outdoor campus landscapes.

**Methods:** This study hypothesizes these associations through application of partial least squares structural equation modeling (PLS-SEM), inputting data from a sample of university students in Malaysia. In the hypothesized model, we examine the degree of restoration that is enjoyed by subjects within landscapes through the effects of these characteristics. Indirect effects of perceived restorativeness via evaluation of mediation effects associated with perception of landscape characteristics and restoration experience are also investigated.

**Results:** Through validation of the measurement model, we find significant positive coefficient paths with adequate predictive abilities in the hypothesized model. Findings suggest the effect of PSD on perceived restorativeness leads to a better explanation of restoration experience. In addition, perceived landscape characteristics of PSD enhance restoration experience in alignment with perceived restorativeness characteristics.

**Conclusions:** Greater effects on restoration experience come through perceived restorativeness that is affected by PSD, which itself is capable of promoting favorable experiences of restorativeness in a green space and facilitating psychological restorative outcomes. The mechanistic effect of emotional regulation implies a distinct role of green spaces in maintaining good mental health and has relevance to public health models that promote independence and well-being through preventative approaches. The work paves the way for further studies that examine which dimensions of PSD support perceived restorativeness and restoration experience more than others, and the wider psycho-social value of green spaces through the application of mediation effects and personal sensory dimensions in the development of mental health.

## Introduction

Mental fatigue and stress-related mental disorders, depression, and anxiety are related to individual mental health and well-known risk factors for positive mental health (1–3). One in every four people suffer from mental disorders in their lifetime (1). Environmental health related researchers have suggested that green environments can reduce levels of stress related mental disorders and therefore enhance positive mental health (2, 4–12). For instance, Dzhambov et al. (6) have shown that green spaces afford restorative experiences and promote better mental health with higher mindfulness, lower rumination, and greater resilience to stress.

In human health and environmental relationships, the positive effect of green spaces on development of positive mental health is recognized as psychological restoration experience (13). Psychological restoration is the direct effect of contact with green spaces to promoting positive mental health outcomes (14). It refers to renewal of “directed attention capacities” that have depleted with sustained usage in daily life, “physiological changes from tension and stress toward relaxation,” and “positive mood change” (15). Examining the literature, a great deal of attention has been paid to restoration experiences in investigating mental health promotion of green spaces (1, 6, 12). The importance of green spaces in the development of better state of mental health involves “the promotion of subjective well-being,” “the prevention of mental disorders” and “the treatment and rehabilitation of people affected by psychiatric disorders” (1). A number of approaches used by researchers to measure the mental health benefits of green spaces involve multidimensional definitions of health outcomes, such as cognitive and emotional restoration (1), attention restoration (16), cognitive functioning (4), and emotional well-being (17, 18). According to the World Health Organization (WHO), health is not an absence of illness, it is a state of physical, mental and social well-being (3). Social and environmental stressors, sustained stress, mental fatigue and negative emotional states cause harmful diseases such as heart disease, type II diabetes and mental illness (1, 13), especially if people neglect recharge their psychophysiological and emotional resources. To cope effectively with everyday life demanding mental disorders, people therefore need to regularly restore the impaired resources from a negative state to their original state.

Topics related to mental health promotion of university students have also shown a light on the restorative potential of outdoor campus green spaces for students' psychological restoration and in development of positive mental health (19, 20). Like other population groups, this subgroup encounters various types of social, academic and environmental stressors, and a lack of psychological restoration can cause depression and mental disorders during what is a sensitive life stage (19). For example, Adams et al. (21) showed that students' poor mental health (depression, anxiety, negative affect) is associated to some extent with a decline in immunity system function and acute infectious illnesses including bronchitis, sinusitis, strep throat and ear infection. To enhance university students' mental health, the importance of outdoor campus green space has been recognized as a potential restorative setting that contribute to their psychological restoration (20, 22), attention restoration (23) and mental fatigue restoration (16). As many studies have shown, in outdoor environments there are possibilities to enjoy a psychological restoration experience and mental health benefits. From this perspective, three sets of research objectives have been developed to show how a physical environment works as a prominent supportive and restorative setting contributing to restoration experience and support development of positive mental health:

(1) A growing body of research based on Attention Restoration Theory (ART), has explored whether the green environment is a restorative setting and to what extent landscape components works as a psychological health resource (24–26). Through this lens, the restorative effects of different types of green environments and the effect of various landscape components on human psychological systems were investigated. Results revealed that an environment with presence of landscape components (mainly greenery and water) is health promoting and provide stress-reducing effects and reduce mental fatigue.

(2) Another group of studies has focused on what perceived qualities of green spaces provide restoration experience. Based on ART, studies have described perceived restorativeness as an environmental condition for psychological restoration experience and better mental health condition (1, 2). The perceived restorativeness involves perception of characteristics of “being away” (freeing the mind from everyday life demanding tasks), “fascination” (effortless attention to pleasing objects like birds and flowers), “extent” (occupying the mind for a long period of time) and “compatibility” (a good match with inclinations, no struggle). Perceived restorativeness characteristics can improve mental health by providing relief from stress related mental disorders (1). For many years, scholars have addressed the questions around psychological restoration experience, cognitive restoration and mental fatigue recovery through examining the impact of these perceived restorativeness characteristics (16, 17, 24, 27).

Although perceived restorativeness is now associated with an extensive literature relating to restorative outcomes in natural environments, in recent decades a number of studies have indicated the possibility of Perceived Sensory Dimension (PSD) of green space for restoration experience and mental health promotion (3, 13, 28–31). PSD involves eight perceived qualities of “nature,” “culture,” “social,” “prospect,” “rich in species,” “refuge,” “space” and “serene.” Within this approach, landscape perception involves active interaction with green space and experience of characteristics such as self-growing lawns (nature), static and dynamic elements offering fascination with the course of time (culture), vistas and plane view (prospect), facilities available for gathering (social), spacious and free (space), diverse non-human life (rich in species), sensation of a safe environment (refuge) and natural sounds offering a sense of quietness and tranquility (serene). It has subsequently been proposed that perception of a green environment with these landscape characteristics offers psychological and emotional restoration from stress (28, 29) and support mental health strengths (13).

(3) The third group of studies went further, and investigated how the “external” environment (green space) is able to affect a human's “internal” being state (18, 32). These scholars believe that the process of understanding how an environment impacts human health is more complex than simply examining what causes the outcome (18, 33). Such studies have focused on the underlying mechanistic factors–mediation effects–and analysis of multiple pathways to explain precisely how this process works. Literature on green space and human health has revealed many possible mediating variables in this relationship (6, 7, 34, 35). Developed from ART, perceived restorativeness is a psychological mechanism that makes it possible to achieve restoration experience in a green environment. For example, Marselle et al. (18) and Hipp et al. (32) examined the mediation effect of perceived restorativeness to explain how perceived naturalness and biodiversity are related to an individual's emotional well-being and quality of life. They showed that an individual's perception of the environment is linked to perceived restorativeness and is in turn a predictor of positive outcomes. In other words, the perception of the environment is associated with positive outcomes, and that outcomes are related to the effects of perceived restorativeness being experienced by subjects in green environments.

A central question nevertheless remains: what is the combined effect of PSD and perceived restorativeness on restoration experience? And more specifically, to what extent are mediating variable(s) of perceived restorativeness decisive in explaining how PSD provides restoration experience? Marselle et al. (18) suggests that the perception of green space qualities *per se* may not be a major factor in understanding how the green environment might affect outcomes. They have pointed out that “… one can look to theories of restorative environments which identify salutogenic outcomes from interaction with, and the qualities of, environments that facilitate well-being” (pp. 218). In fact, they have suggested that while perceived restorativeness may play a mediating role in the impact of perception of environmental qualities on emotional well-being, the mediation effects of PSD and restoration experience remain unexplored in the literature. In testing pathways linking green space to health, the modeling approach introduce the methodology of multivariate relationships and sensitive analysis of indirect effects, which few studied followed that (1, 20, 35).

The aim of this study therefore is to systematically examine the supportive and restorative qualities of campus green space that promote mental health on the psychological restoration of a sample of university students. It considers the process of restoration experience through systematically examining the effects of PSD and perceived restorativeness. A partial least squares structural equation model (PLS-SEM) is employed to simultaneously examine the degree of associations between PSD, perceived restorativeness and restoration experience, and more particularly test whether perceived restorativeness can play a mediating role between PSD and restoration experience.

The remainder of the paper is organized as follows: the subsequent four sections conceptualize the various relationships between restoration experience, perceived restorativeness and PSD and potential mediation effect variables. The purpose is to inform development of a conceptual model which frames the hypotheses to be tested. Research methods are then described and statistical analysis and results are presented around three main axes: a measurement model, a structural model and mediation effects. Findings are discussed in relation to the hypotheses, existing literature and the implications for green space mental well-being and wider psycho-social interventions together with associated landscape design considerations.

## Literature Review

### Relationship Between PSD and Restoration Experience

Restoration experience has mostly been discussed with reference to two theories–Stress Restoration Theory (SRT) and ART (13). Based on SRT, restoration in contact with nature derives from stress recovery. It can be manifested through beneficial changes in emotional states and in activity of physiological dimensions of stress response such as blood pressure, heart rate and muscle tension. Based on ART, contact with a natural setting improves psychological resources e.g., capacity of direct attention, mental recovery and cognitive ability. Related studies have supported different aspects of restoration experience in contact with nature (13, 16, 17, 24). Based on these fundamental theories (15), restorative outcomes of natural environments have been operationalized into dimensions of “direct attention restoration,” “clearing random thoughts” and “relaxation and calmness”. Based on the literature, these outcomes provide the foundation for explaining an individuals' perceived state of restoration in the green environment (15, 17, 36). In this paper, we address restoration experience (outcomes) in outdoor campus landscapes in terms of these three dimensions.

Specification of green spaces characteristics are the result of several attempts between the years 1985 to 2010 (27, 28). Based on Salutogenesis' concept and Supportive Environment Theory (SET), three sets of characteristics have been suggested and PSD is the outcome of a third generation of these attempts (13). Grahn and Stigsdotter (28) generated PSD through a factor analysis of the preference ratings of 953 individuals (Swedish population) from a long list of green space experiences. The aim was to identify preference for landscape characteristics that offer restoration from stress and thus improve mental health. The idea in development of PSD is based on Gibson's ecological approach to perception. Within this perspective, landscape perception is the consequence of a perception-action process involving movements of the entire body, stimulation and combination of all sensory systems (28). Landscape perception based on this approach facilitates detection of open space qualities for restoration experience and maintains mental health through collaboration of multiple senses (i.e., touch or tactile sensation, hearing, sight, and smell).

Many studies have recommended that PSD is a supportive requirement to reduce stress, achieve psychological restoration and maintain mental health strength (3, 29, 30, 37, 38). For instance, Memari et al. (13) showed that experience of PSD has created environments offering mental restoration through investigating four dimensions of stress recovery: emotion, physiology, cognition, and behavior and that support people mentally and physically in their everyday life. In the evaluation of mental health promotion of a forest designed environment (29) the presence of PSD components were shown to elicit more restorative responses by measuring psychological restorativeness in regard to providing stress relief. The study (28) also indicated the importance of PSD to public mental health with respect to restoring people from stress. With reference to a nature-based rehabilitation experience (31) showed that PSD is supportive for mental recovery of individuals with stress-related mental disorders. Similarly, Stoltz et al. (38) showed that PSD is a relevant factor with regard to stress reducing in planning of the restorative forest environments to improve human lives and health. Topics relating to mental health promotion of PSD lack research that addresses the importance of perceived sensory qualities of green spaces in relation to psychological restoration experience. When people are exposed to green environments with the presence of perceived characteristics of PSD, it may contribute to their psychological restoration in terms of dimensions, as operationalised by Korpela et al. (15). Thus, the following hypothesis is proposed:

Hypothesis 1: The association between PSD and experience of psychological restoration in green space–in terms of restorative outcomes of “direct attention,” “random thoughts” and “relaxation and calmness” –is positive.

### Relationship Between Perceived Restorativeness and Restoration Experience

The concept of perceived restorativeness is drawn from ART (39). It divides human attention into two parts as direct attention (voluntary form of attention) and indirect attention (involuntary attention). The direct attention is under intentional control, a finite resource and can easily be depleted with high usage (40). Sustained use of direct attention causes exhaustion and leads to Directed Attentional Fatigue (DAF) or so-called mental fatigue. DAF decreases effectiveness in functioning (e.g., less ability to work without error), causes a variety of negative emotions (e.g., bad humor, irritability, impatience) and eventually leads to serious health related problems (40). In contrast to direct attention, which is susceptible to mental fatigue, indirect attention is resistant to fatigue. It is an effortless process with unlimited capacity. Based on ART, a physical environment with presence of restorative characteristics has the ability to recover mental fatigue or diminish psychological resources (41). In fact, the mechanistic effect of perceived restorativeness is the actual requirement in this process, which involves perception of “being away” (escape and novelty), “fascination,” “extent” and “compatibility” that can lead to psychological and cognitive restoration experience (42, 43). Studies have consistently supported the positive relationship between these restorativeness characteristics and measures of restoration experience. Along with an increase in perceived restorativeness they can lead to an increase in sustained direct attention, stress relief and recovery of mental fatigue (16, 24). For instance, in examining mental health promotion of physical environments, Martínez-Soto et al. (1) showed the recovery of cognitive and affective resources when people are exposed to environments with perceived restorativeness characteristics. In short, perception of restorativeness characteristics enhance the potential of a natural setting for building psychological resilience and promoting better mental health state (6).

There are two approaches to the usage of perceived restorativeness evident in the literature. Some use it as an instrument to measure psychological restoration experience (44, 45), while others use it to measure the capacity of restorativeness of a green environment (16, 46). Although the characteristics involved in perceived restorativeness and restoration experience emphasize the recovering aspects of green environments (47), they are not the same and measure different types of effects (48). Perceived restorativeness is a subject's perception of the restorative capacity of a green environment and restoration experience is the degree to which a subject realizes beneficial changes in contact with a green environment (47, 48). The observed difference that exists between the two measures leads us to examine this relationship further and develop the following hypothesis:

Hypothesis 2: The association between perceived restorativeness and experience of psychological restoration in green space is positive.

### Relationship Between PSD and Perceived Restorativeness

A number of researchers have shown that there is association between perception of green environments and perceived restorativeness (1, 10, 49). From a mental health perspective, the measure of perceived restorativeness takes into account perception of green space qualities that may have substantial restorative value in capturing psychological benefits. For instance, Lai et al. (41) showed that pleasantness and aesthetic quality are significant predictors of perceived restorativeness. Several other perceived qualities have been investigated in this association such as comfort, safety, attractiveness and maintenance (14). However, Grahn and Stigsdotter (28) suggest that perceptions of green space in relation to restoration experience and the improvement of mental health also involves other substantial multi-sensory qualities, otherwise known as PSD. It has been claimed that PSD can be useful for the evaluation of perceived restorativeness of green spaces and to help develop tools for practitioners in the planning and design of restorative settings to improve mental health (27, 30). To the best of our knowledge, the study by Peschardt and Stigsdotter (27) is the only one to show an association between PSD and self-perceived restorativeness in public urban green spaces. The authors evidence this relationship through examining seven out of eight PSDs and undertaking an expert assessment of PSDs. While the expert assessment is useful (50), there are some conditions that require the users' perspective (51), particularly when determining the results of direct interaction with environment on their health and well-being (28).

The user's experience and active perception of green environment qualities is an important aspect in the development of PSD (28). The expert objective measure of PSD does not take into account all the experiences that determine a user's perception of the green environment (51). Human perceptions and experiences are more likely to be influenced by individuals' characteristics and health status (50) and provide different outcomes, even comparable environments with similar characteristics (45). Thus, the present study seeks to test this relationship according to a green spaces' user's perspective. This is particularly important for determining the ability of a green space to affect a human's being state. Therefore, the following hypothesis is proposed:

Hypothesis 3: The association between PSD and perceived restorativeness from a user's perspective is positive.

### Drivers of Mediation Effects

A mediator variable theoretically links a predictor variable to a criterion variable to provide more information about the relationship (52). Several studies have shown the significant mediating effect of behavioral variables such as physical activity, social cohesion, loneliness (53) and social contacts (54) in the relationship between a green environment and human health. In addition to mental health *per se* this highlights the significance of green spaces for a range of psycho-social outcomes (55, 56) associated with the wider social value of such environments. It also highlights the importance of mediator variables and associated modeling approaches for understanding, evaluating and predicting the experience of psycho-social outcomes as a result of spending time in green environments. Some studies have reported a weak mediating effect, i.e., spending time in nature in the relationship between the level of residential greenness and residents' mental health (57). However, in the literature, there is a general agreement that the perceived restorativeness is a psychological mechanism that underlies the relationship between natural environments and perceived positive health related outcomes such as likelihood of restoration (24), quality of life (32), emotional well-being (18, 46) and a reduction in anxiety and depression as important components of better mental health (6).

Although, the mediating factors increasingly reveal possible reasons how nature affects human health, little research has been conducted on the mediating effect of perceived restorativeness in the relationship between PSD and restoration experience. Most PSD studies have indicated which perceived dimensions of PSD are more optimal for restoration from stress (3, 13) or restorativeness experience (27). However, very few aim to understand whether perceived restorativeness plays a mediating role in the relationship between PSD and restorative outcomes. In addition, whether perceived restorativeness is the only underlying mechanism in this relationship is considerably less clear. Operating mechanism/s help to indicate how effective PSD is for the generation of restoration experience in green spaces. Therefore, the following hypothesis is proposed:

Hypothesis 4: The relationship between PSD and restoration experience is positively mediated by perceived restorativeness.

### Hypothesized Model

We undertake examination of all four research hypotheses using PLS-SEM as the statistical modeling technique. PLS-SEM is a prediction-oriented method with an exploratory approach and estimates multiple variables, at the same time. It is especially applicable when circular relationships or loops are not allowed in the model and provides a statistical statement about the relationships between a set of theoretically established variables, which are measured in a quantitative fashion (58).

**Figure 1:**
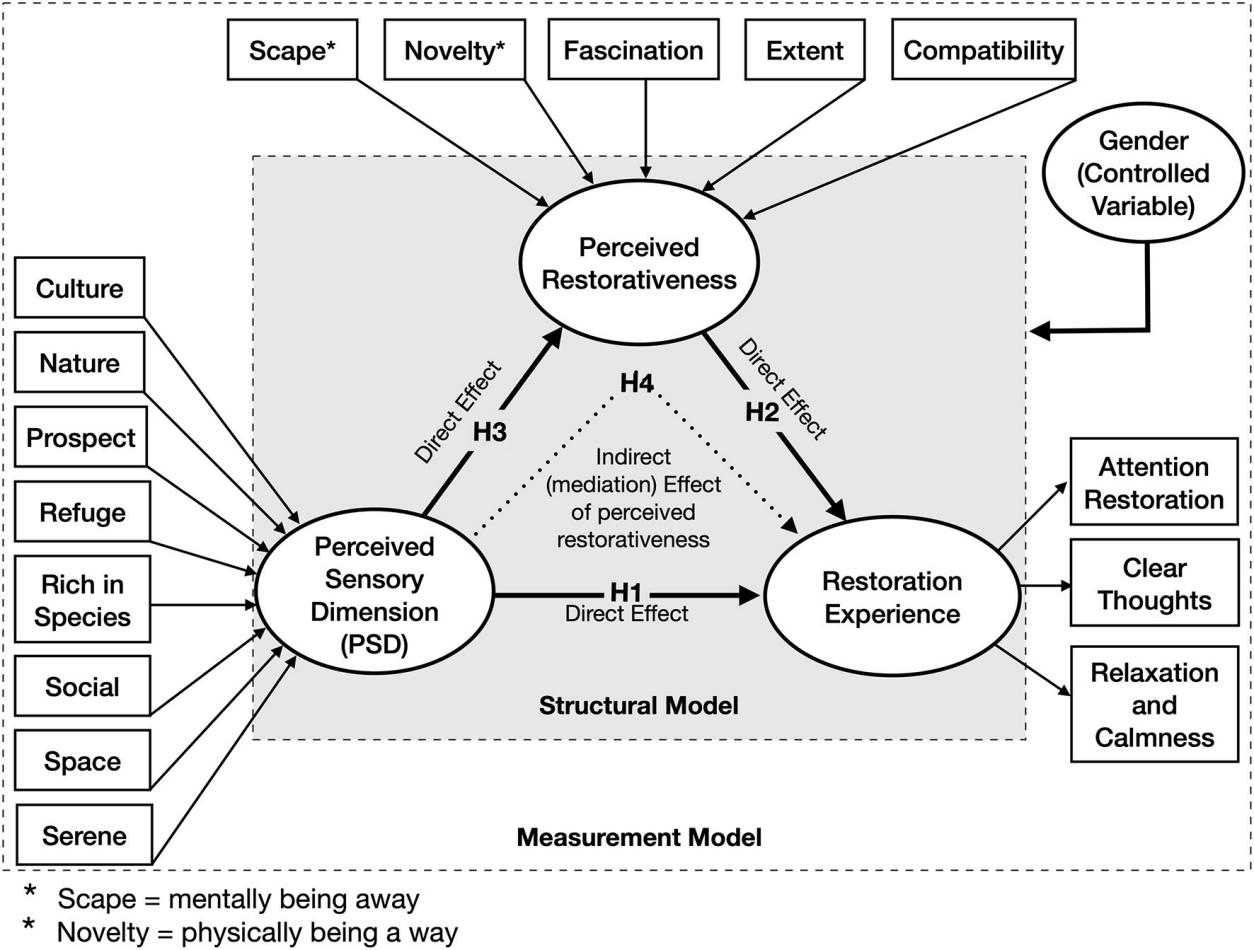
Conceptual framework.

The conceptual framework outlined in Figure 1 is based on the proposed hypotheses. The path model involves two main elements–a structural model and a measurement model. The structural model contains the hypothesized relationship between constructs that have been developed from the underlying theories and concepts. And as its name depicts, the measurement model contains measurement variables or indicators. As the framework indicates, the study focuses on the relationship between PSD and restoration experience (H1), the relationship between perceived restorativeness and restoration experience (H2) and the relationship between PSD and perceived restorativeness (H3). In addition, it tests the mediation effect of perceived restorativeness on the relationship between PSD and restoration experience (H4). In the PLS-SEM path model, restoration experience is a reflective variable while PSD and perceived restorativeness are formative variables. In the reflective model, “the measures are all caused by a single underlying construct,” and in the formative model “the measures all have an impact on (or cause) a single construct” (59). Therefore, in the reflective model, the direction of the arrows is from restoration experience through to its measurement indicators and in the formative models, the arrows are from the measurement indicators through to their constructs (PSD and perceived restorativeness). In this model, we used gender as a control variable to test its effect on the model evaluation.

## Research Methods

### Sampling and Sample Size

**Figure 2:**
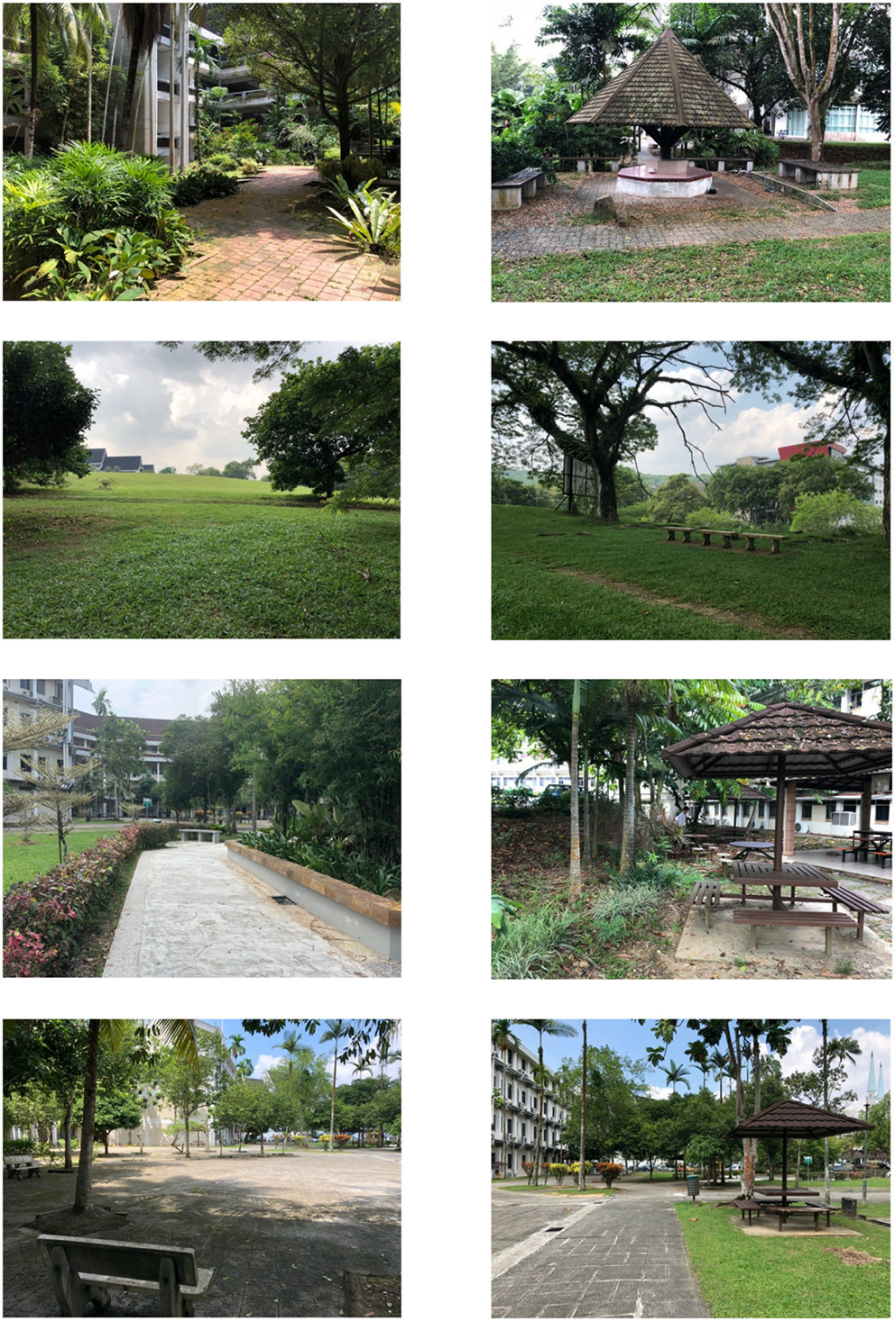
Some images of University outdoor green spaces.

This study, involving a sample of university students in Malaysia, tested a theoretically derived model of restoration experience through the impact of PSD and perceived restorativeness. Students from the Universiti Kebangsaan Malaysia (UKM), University Malaya (UM), Universiti Putra Malaysia (UPM), Universiti Sains Malaysia (USM) and Universiti Teknologi Malaysia (UTM) were taken as the study population. Data were collected through self-completion surveys administered in lecture rooms in each university, in the middle of 2014–2015/2 academic session. This time was selected to ensure that the majority of the students had ample opportunities to use green campus outdoor spaces for recreation. As respondents were required to assess perceived outdoor green space qualities as well as after-visit restorative outcomes, only students who had frequently engaged in use of outdoor campus green space recreation (within 2 months) were requested to answer the survey. This criteria was informed by a previous study of the perceptions of campus green space for students' restoration experience (32) and recalled restorative outcomes of the most recent visits to green environments (17). Through this criterion, we excluded those students that just passed the campus green spaces on their way to other locations. Because, for example, when students passed campus green spaces by bus or car, they were unlikely to be able to transfer the information that is required for assessment of sensory dimension of green qualities and attribution of qualities to outcomes. The green outdoor space in these universities include landscapes around the academic buildings and the overall campus (see Figure 2).

A total of 550 students participated in the study. In a data screening procedure, those with missing data, suspicious response patterns and outliers were excluded (*n* = 106) yielding 444 usable responses for the analysis. The response rate was 81%, with the sample consisting of 300 female and 144 male students. The majority of them were single (93.24%), undergraduate (79.73%), native (92.12%), studying full-time (94.59%), living on a campus setting (77.70%) and ranging in age from 19 to 30 years. The over-representation of females in the sample compared to the wider population was due to their willingness to participate in answering the survey. However, no significant differences were found between the responses of male and female subjects.

### Measurement of Variables

The Restorative Outcome Scale (ROS-6 items) was used in the measurement of restoration experience, as applied in previous studies on the perceived restorative outcomes in a visit to green environments (15, 17, 36). This scale subjectively measures restorative outcomes of the visit to a green environment in terms of recovery in the “direct attention restoration” (1 item), “clearing random thoughts” (2 items) and “relaxation and calmness” (3 items). This scale was measured on a 7-point scale from “not at all” through to “completely.” The mean value of the items was used to indicate the variable value. Although three dimensions of “direct attention restoration,” “clearing random thoughts” and “relaxation and calmness” have been proposed, the factor analysis showed only one factor loading (15). Because of this reason, the three variables of “direct attention restoration,” “clearing random thoughts” and “relaxation and calmness” have been presented as reflective indicators of the construct of restoration experience. The reported Cronbach's alpha was 0.92 in previous studies (15).

The Restorative Components Scale (RCS-22 items) was adopted to measure perceived restorativeness (42). We measured “novelty” with three items, “escape” with four items, “fascination” with six items, “extent” with four items and “compatibility” with five items. As Laumann et al. (42) explains, in the development of this scale, two concepts emerged from the original term of “being-away.” Novelty–which is a physically being-away, and Escape–which is mentally being away in a green environment. In ART, both physically and mentally being-away aspects of the natural environment are essential conditions for perceived restorativeness (60). The item development in this scale did not emphasize the aspects that make their usage specific for a population group or a particular environment (42). Again, question responses in this section were measured on a 7-point scale from “not at all” through to “completely.” The psychometric properties of this scale have been previously reported by Laumann et al. (42) and Cronbach's alpha for the subscales ranges from 0.76 to 0.86. Mean responses for each variable of perceived restorativeness were used.

Measures of PSD were adopted from items used in the study by Grahn and Stigsdotter (28). Prior to final data collection, we carried out an item verification step (with a group of university students and staffs) to rate the most relevant items–from the list of 65 items identified in Grahn and Stigsdotter (28)–to the context of campus green space (from 1 “not relevant” to 4 “highly relevant”). For each of the eight perceived dimensions of PSD, we considered the most three commonly rated items. This yielded a total number of 24 items for use in the final survey, again all on a 7-point scale from “totally disagree” through to “totally agree.” We used mean responses for each dimension of PSD, which has previously been applied in the measurement of green space characteristics across different cultural backgrounds and contexts, such as forest environments, urban parks and care settings (13, 29). Previous studies provided support for the reliability and validity of using PSD in the measurement of green space characteristics (28, 29, 51). Indeed, it has been shown by Memari et al. (13) that all the variables of PSD (except Refuge) had acceptable or good Cronbach alpha values (0.7). The variable Refuge, although showing lower Cronbach alpha in the present study, was considered as acceptable and was duly used. The PSD measurement in this research was based on using four items for each dimension.

In addition, following Hair et al. (58), we included two single-item questions for convergent validity of PSD and perceived restorativeness in the PLS- SEM formative measurement model. These questions asked students to rate their overall perception of restorativeness in the visited site and the sensation of being in a green space with greenery characteristics (from 1 = “not at all” through to 7 = “completely”).

## Results

SmartPLS version 3.0 was used to analyse the data (61). We followed suggested evaluation steps for the PLS-SEM analysis by Hair et al. (58). These steps comprise first, an assessment of the measurement model (formative and reflective), followed by the structural model and finally the meditational model. Results from these analyses are described and presented in the following sections.

### Reflective Measurement Model: Restoration Experience

The reliability and validity tests of the reflective measurement model, were conducted by internal consistency reliability, indicator reliability, convergent validity and discriminant validity (Table 1). The Cronbach's alpha exceeded the threshold value (= 0.861 > 0.7) and the composite reliability value is 0.915, which is within the required range (>0.7 and <0.95). The outer loadings of measurement indicators are well-above the required minimum level of 0.708. Acceptable values in measures of the inter correlations of measurement indicators and outer loadings of indicators demonstrate a high level of internal consistency and indicator reliability for construct of restoration experience in reflective measurement model in PLS-SEM. The summary statistics for this variable is presented in Table 1.

**Table 1:** Evaluation of validity and reliability of restoration experience in reflective measurement model.

	**Discriminant validity**									
	**Cross loadings**			**Fornell-larcker criterion**			**Indicator reliability**	**Internal consistency reliability**		**Convergent validity**
**Indicators and constructs**	**PR**	**PSD**	**RE**	**PR**	**PSD**	**RE**	**Outer loading**	**Cronbach's alpha**	**Composite reliability**	**AVE**
Attention restoration	0.557	0.451	0.892	-	-	-	0.892	0.861	0.915	0.781
Clearing thoughts	0.583	0.474	0.883	-	-	-	0.833	-	-	-
Relaxation and calmness	0.661	0.553	0.877	-	-	-	0.877	-	-	-
PR	-	-	-	-	-	-	-	-	-	-
PSD	-	-	-	0.655	-	-	-	-	-	-
RE	-	-	-	0.562	0.684	0.884	-	-	-	-

*Summary statistic restoration experience = Attention restoration (“Min, 1,” “Max 7,” “Mean 5.65,” “STD 1.17”), Clearing Thoughts (“Min, 3,” “Max 7,” “Mean 5.57,” “STD 1.02”) and Relaxation and Calmness (“Min, 3,” “Max 7,” “Mean 5.35,” “STD 1.04”)*.

*PR, Perceived Restorativeness; PSD, Perceived Sensory Dimension; RE, Restoration Experience*.

The result of AVE is 0.781, which established that the indicators explain almost 80% of the variation in restoration experience. The discriminant validity shows how much a variable is unique and captures concepts that are not presented by other variables in the model. In a cross-loading assessment, the outer loading of each measurement indicator of restoration experience is higher than all of its cross loadings on the perceived restorativeness and PSD. In the Fornell-Larcker criterion, the square root of AVE of restoration experience should be greater than its highest correlation with perceived restorativeness and PSD. The square root of the VAF value for restoration experience is 0.884, which is higher compared with its correlation with PSD and perceived restorativeness, implying satisfactory discriminant validity.

### Formative Model: PSD and Perceived Restorativeness

The summary statistics and reliability of the scales for each variable in PSD and perceived restorativeness are presented in Table 2. As shown, for some of the variables the Cronbach Alpha is less than the proposed 0.7 (62) using the mean inter-item correlation when the number of items are low in the reliability assessment. For all variables where the value of Cronbach's Alpha are <0.7, the mean inter-item correlation should be between 0.2 and 0.4 as directed by Briggs and Cheek (63). The internal consistency of most of the PSD and perceived restorativeness characteristics are thus within the required range.

**Table 2:** Summary statistic and Cronbach's alpha of all variables (PSD, Perceived Restorativeness and Restoration Experience).

**Indicators**	**Min**	**Max**	**Mean**	**STD**	**Cronbach's alpha**	**Inter-item correlations**
Culture	2.33	7	4.88	0.98	0.554	0.294
Nature	2	7	5.17	1.04	0.674	0.408
Prospect	2	7	5.15	0.99	0.646	0.378
Refuge	2.67	7	5.06	0.91	0.468	0.227
Rich in species	1	7	4.5	1.3	0.855	0.664
Social	2.67	7	5.41	0.84	0.528	0.278
Space	1.67	7	5.22	0.89	0.517	0.265
Serene	2	7	5.32	1.09	0.540	0.322
Escape	2.50	7	5.00	0.99	0.743	0.421
Novelty	2.33	7	4.97	0.99	0.550	0.389
Fascination	2.6	7	5.02	0.90	0.828	0.446
Extent	3	7	5.07	0.86	0.752	0.432
Compatibility	2.4	7	5.05	0.88	0.808	0.460

The formative measurement model was evaluated through convergent validity, the presence of collinearity among indicators and the significance and relevance of indicators. To examine convergent validity of PSD and perceived restorativeness a simple model connecting the correlation of indicators of each formative variable to its single-item global reflective measure of that variable was specified. The path coefficient in PSD is 0.836 and *R*^2^ value is 0.699. For perceived restorativeness, the path coefficient is 0.839 and *R*^2^ value is 0.704. These results show that the reflective measure of PSD and perceived restorativeness are highly and positively correlated with their formative measurement indicators, which indicate validity of the formative measurement model (higher than 0.7 and *R*^2^ > 0.5).

The Variance Inflation Factor (VIF) in Table 3 shows that there is no high inner correlation between a measurement indicator and the remaining indicators that are associated with PSD and perceived restorativeness. The value below 0.5 shows the indicators to be highly correlated with their variables, thus there is no collinearity issue in the formative measurement model. Bootstrapping (10,000 sub-samples) is used to establish significant outer weights to estimate the absolute importance of each formative indicator in the measurement of its variable without considering any other indicators. The non-significant indicators should be removed from the model when the theoretical background supports this decision. Alternatively, the outer loadings must be above 0.50. As demonstrated, all indicators of the perceived restorativeness construct are shown to be significant (*t*-value > 1.96). In PSD, the “culture” and “refuge” are insignificant. When checking the outer loadings, we find these indicators to be of relative importance for the explanation of PSD in the model, given by the satisfactory outer loadings for “culture” 0.507 and for “refuge” 0.716. Overall, the combination of desirable values provides significant evidence for the formative measurement model validity. These results show the model to contain the entire domain of theoretically derived variables for hypotheses testing, making the case for assessment of the structural model.

**Table 3:** Evaluation of validity and reliability of PSD and perceived restorativeness in formative measurement model.

**Indicators**	**VIF**	**Std. Dev**.	***t*-test**	***p*-value**	**Outer weights**	**STD**	***t*-test**	***p*-value**	**Outer loadings**
Culture	1.558	0.070	1.064	0.287	−0.071	0.071	1.045	0.296	0.507
Nature	1.908	0.072	2.821	0.005	0.196^**^	0.072	2.829	0.005	0.729^**^
Prospect	1.554	0.074	3.347	0.001	0.249^**^	0.074	3.324	0.001	0.709^**^
Refuge	1.852	0.073	1.551	0.121	0.121	0.072	1.567	0.117	0.716
Rich in Species	1.896	0.074	3.057	0.002	0.234^**^	0.073	3.095	0.002	0.659^**^
Social	1.164	0.056	2.833	0.005	0.156^**^	0.057	2.784	0.005	0.342^**^
Space	1.378	0.070	3.739	0.000	0.255^***^	0.07	3.74	0.000	0.667^***^
Serene	1.368	0.065	5.687	0.000	0.367^***^	0.065	5.762	0.000	0.686^***^
Escape	1.635	0.052	5.076	0.000	0.185^***^	0.052	3.552	0.000	0.579^***^
Novelty	1.286	0.043	3.534	0.002	0.134^**^	0.043	3.182	0.001	0.485^**^
Fascination	1.589	0.059	4.131	0.000	0.458^***^	0.059	7.803	0.000	0.869^***^
Extent	1.699	0.055	7.818	0.000	0.226^***^	0.055	4.171	0.000	0.738^***^
Compatibility	1.195	0.065	3.146	0.000	0.332^***^	0.065	5.085	0.000	0.791^***^

*Significance levels*:

** *ρ < 0.01*,

*** *ρ < 0.001*.

### Structural Model: Significance of Relationships and Model Predictive Abilities

This step was performed by assessing the structural model for collinearity issues, assessing the significance of structural model relationships, and assessing the predictive abilities of the model according to the Coefficient of Determination (*R*^2^ value), effect size *f* ^2^ and *Q*^2^ and blindfolding. First, we assessed if collinearity among predictive variables was an issue in the structural model. The PSD is shown to be a predictor of restoration experience and perceived restorativeness; and perceived restorativeness to be a predictor of restoration experience. Table 4 shows the VIF values are higher than 0.20 and lower than 5.00, indicating significant levels of collinearity in the structural model.

**Table 4:** Structural model evaluation-significance and predictive abilities of constructs relationships.

**Constructs**	**VIF**	***t*-values**	***p*-value**	**Path coefficients**	***R*^**2**^ value**	***F*^**2**^ value**	***Q*^**2**^ value**	**Hypotheses**
PSD to RE	1.751	4.310	0.000	0.197^***^	-	0.043	-	Supported
PSD to PR	1.000	25.276	0.000	0.656^***^	-	0.753	-	Supported
PR to PSD	1.751	13.423	0.000	0.555^***^	-	0.344	-	Supported
PR	-	0.034	0.000	-	0.430^***^	-	-	-
RE	-	0.034	0.000	-	0.485^***^	-	0.356	-

*Significance levels*:

*** *ρ < 0.001*.

*PR, Perceived Restorativeness; PSD, perceived Sensory Dimension; RE, Restoration Experience*.

We then assessed the significance of relationships among the variables, using the PLS algorithm and Bootstrapping. The three paths are significant with *t*-values greater than the threshold value of 1.96 (0.197^***^, 0.656^***^, and 0.555^***^). With respect to the models' predictive ability, the Coefficient of Determination (*R*^2^ value) of 0.430 and 0.489 can be considered a moderate level of predictive accuracy (58). The effect size of f^2^ shows that a large effect of PSD on perceived restorativeness (0.753) is evident, together with a medium effect of perceived restorativeness on restoration experience (0.344) and a relatively small effect of PSD on restoration experience (0.043). The value of *Q*^2^ is considerably greater than the threshold limit (0.356 > 0), which implies the predictive relevance of restoration experience in the model. None of the constructs are found to have a very small predictive power. In the structural model evaluation, the results show the significance and predictive relevance of relationships, and so support the three research Hypotheses; H1, H2, and H3. With the significance of relationships between variables in the structural model established, the suggested mediator variable on the relationship between PSD and restoration experience was then tested.

We used gender as the control variable for this study. The result showed that the difference in effect of PSD on restoration experience between male and female was 0.065 (*p*-value = 0.721), PSD on perceived restorativeness was 0.004 (*p*-value = 0.461) and restoration experience to perceived restorativeness 0.028 (*p*-value = 0.380). Since there were no significant differences between the two groups of male and female in the model evaluation, we can say with some confidence that the control variable gender does not affecting relationships between PSD perceived restorativeness and restoration experience.

### Meditational Model Evaluation

In the mediator model, we examined if the (theoretically established) direct relationship between PSD and restoration experience is mediated by the indirect effect of perceived restorativeness. There are several steps in conducting the mediator effect analysis in PLS-SEM (58). In the first phase, it assessed the direct effect, which is the impact of PSD on restoration experience directly without including the mediator effect of perceived restorativeness in between. If the path coefficient of this relationship is significant (*p*-value < 0.05 and T Statistic > 1.96), then the second step will take place, otherwise there is no mediation effect when the original cause and effect relation is insignificant. As shown by the data in Table 5, the path coefficient is significant (0.569^***^ with a *t*-value of 17.628).

**Table 5:** Mediation effect of perceived restorativeness.

**Constructs**	**Direct effect**	***t*-value**	***p*-value**	**Indirect effect**	**Total effect**	**VAF**
PSD to RE (without mediation)	0.569^***^	17.628	0.000	-	-	-
PSD to RE (with mediation)	0.197^***^	4.310	0.000	0.362	0.562^***^	64.9%
PR to RE	0.555^***^	13.423	0.000	-	-	-
PSD to PR	0.656^***^	25.276	0.000	-	-	-

*Significance levels*:

*** *ρ < 0.001*.

*PR, Perceived Restorativeness; PSD, perceived Sensory Dimension; RE, Restoration Experience*.

The next step is to examine the indirect and total effect. The indirect effect is the impact of one construct (PSD) on another one (restoration experience) through examining the effect of an intermediate construct (perceived restorativeness). Combining the direct and indirect effects creates the total effect, which shows the overall impact of one construct on a dependent construct. The effect should be significant and the mediator absorb some of the direct effect to demonstrate mediation in the model. Both paths from PSD to perceived restorativeness and from perceived restorativeness to restoration experience have a significant value. The path coefficient for indirect effect of PSD on restoration experience is 0.362 and the total effect value is as high as 0.562. With inclusion of the indirect effect of perceived restorativeness, the indirect effect is absorbed the PSD's effect on restoration experience. The direct effect becomes smaller (0.569^***^ to 0.197^***^) when the effect of perceived restorativeness is included, confirming the presence of a mediator effect in the model, and supporting Hypothesis 4.

Finally, the variance accounted for (VAF) was calculated to determine how much the mediator variable absorbs the direct relationship. It determines the extent to which the variance of restoration experience is directly explained by PSD and how much the target's construct variance is explained by the indirect effect of perceived restorativeness. There is no mediation when the VAF is <20%, “partial mediation” when the VAF is larger than 20% and <80% and a “full mediation” effect occurs when VAF is above 80%. In this case, the value of VAF confirms that 64.9% of PSD's effect on restoration experience is explained via the perceived restorativeness mediator.

## Discussion

The influence of green space on restoration experience and mental health is a longstanding research topic. In this respect, there is a growing interest on the important role of perceived qualities that can be linked to restoration of capacities and enhance of mental health benefits. Studies have shown that in green environments there are characteristics that support people mentally in their everyday life. Outdoor campus green space is such an environment that include landscapes with greenery qualities with the potential to provide a variety of positive outcomes in support of the university students' mental health. However, relatively little attention has been paid to examining relationships between perceived green space characteristics and restoration experience, or to investigating the mediation effect variables that explain how a green space affects beneficial outcomes.

The aim of this study was to test a theoretically developed model of restoration experience through the effects of PSD and perceived restorativeness. Moreover, we hypothesized that the relationship between PSD and restoration experience is positively mediated by perceived restorativeness. Through employment of a PLS-SEM modeling technique, we have tested the direct and indirect relationships and demonstrated a significant association between PSD and perceived restorativeness with restoration experience. With this model, we have been able to describe almost 50% of the variance in restoration experience and demonstrate that PSD is capable of offering a psychological restorativeness experience, which is an essential step for better explanation of restoration experience. Examination of perceived restorativeness and PSD and psychological restoration provide evidence-based design recommendations for university campus settings in students' mental health promotion. Related observations, including the contribution to existing evidence and suggestions for future research, are discussed in the following sections.

### Relationship Between PSD and Restoration Experience

Previous studies have discussed the restorative value of characteristics of PSD for stress-related mental disorders (3, 31) and support of mental health (13, 30). To our knowledge, no studies have yet examined the direct relationship between PSD and restoration experience. The current study addresses this gap and demonstrates this relationship in terms of the restorative outcomes of direct attention, clearing random thoughts and relaxation and calmness. Consistent with the findings of Grahn and Stigsdotter (28), this result indicates that experiences of PSD provide possibilities for restoring people's health, well-being and staying mentally healthy. With regard to restoration from stress, PSD is a key element in the design and implications of health-promoting environments (13, 28, 29) and an important therapeutic factor in nature-based rehabilitation programmes (31). Perception of outdoor campus landscapes plays an important role in the university students' quality of life (32), attention restoration (23) and in meeting of their health needs (10, 19, 20). Identifying environmental characteristics of outdoor campus green spaces by determining multi-sensory experiences of PSD with restorative potential provide recommendations for students' mental health in the context of a university campus. Together with the mediation effects discussed below, PSD has associated relevance to the capture and measurement of psycho-social outcomes as the wider social value of such environments is increasingly recognized.

### Relationship Between Perceived Restorativeness and Restoration Experience

It is also important to evaluate the restorative quality of an environment in order to discuss psychological restorative benefits. In the current study, we hypothesized that perceived restorativeness is directly related to perceived restorative outcomes, as previously examined by Korpela et al. (15). Our data supports this hypothesis. Indeed, a majority of other studies also support it, particularly when demonstrating the role of perceived restorativeness in reducing stress disorders or mental fatigue (16, 24). Based on ART, in a restorative setting, there exist positive features that attract people, hold their attention, draw their thoughts away from external demands or afford them intended activities. These restorative properties are environmental conditions that induce a positive state in an individual's psychological system (60). The restoration experience happens through spending sufficient time in a green environment combined with an experience of its restorativeness qualities (39, 43). Four progressive levels of restoration experience in the green environment are “cleaning the head,” “recharging directed attention capacity,” “enhanced cognitive functioning” and “reflections on one's life.” The final level is the deepest and can be experienced through an increase in subjective vitality and self-confidence. This level requires repeated experiences of restorativeness in green environments (43). The existing research within the health-promoting effect of outdoor campus green spaces suggest that the campus restorativeness has potential (19, 20). Enhancing students' perceived restorativeness by restorative resources on campus can balance multidimensional stress and facilitate psychological restoration from prolonged mental efforts (19). The present findings within the area of university students' mental health development indicate that restoration experience in outdoor campus green space can be enhanced by perceived restorativeness characteristics.

### Relationship Between PSD and Perceived Restorativeness

This study has established a significant relationship between PSD and perceived restorativeness, with PSD including values associated with how people experience and perceive landscape characteristics in green spaces such as calmness and observing several animal and bird species (28). Indeed, according to Grahn and Stigsdotter (28), the experience of green space, especially in relation with mental health support involves perceived sensory systems', for example how users of green spaces enjoy the views, sounds and smells of these environments. Our findings reinforce these observations. In landscape assessments, it is thus important to consider the qualities that people prefer over others when actively seeking a restorative environment (28).

Previously, Peschardt and Stigsdotter (27) used an expert on-site approach to assess park characteristics, in terms of representation of PSD qualities, and found its significant association with the park user's perceived restorativeness. However, Qiu and Nielsen (51) have questioned such approaches as they do not involve users' experience, ideas and feelings. In this paper we therefore tested- and confirmed–this relationship through subjective assessments of PSD. Consistent with previous studies (28, 51), the representation of PSD in green spaces should include people's experiences and sensory perceptions of landscape characteristics. The expert judgements are suitable, particularly for readily monitored attributes such as size, distance and habitat types (51). The self-assessment of PSD enables identification of such qualities that are popular and important in relation to recovery of stress and the support of mental health. Understanding how people actually experience and perceive the qualities of green spaces helps to improve knowledge on exactly which qualities satisfy restorativeness experiences (28, 29). In design and planning for mental health promotion within the area of campus settings, perception of outdoor campus landscapes are found to be crucial factors that contribute to the perception of campus restorativeness. These findings provide new insights into the perception of campus qualities for restoration and support the results of previous research regarding perceived qualities and campus restorativeness (23, 32).

### Mediation Effect of Perceived Restorativeness

In Marselle et al. (18) and Hipp et al. (32)'s meditational models, the perception of environmental qualities was presumed to cause perceived restorativeness. Conversely, perceived restorativeness was deemed to result in improved emotional well-being and quality of life. In this paper we have built on these studies, and established the same positive conclusion that perceived restorativeness provides restorative outcomes, and plays a mediation role in the relationship between PSD and restoration experience. While significant, perceived restorativeness was found only to be a partial mediator of the effect of PSD on restoration experience. This finding is similar to previous findings (32) that have found perceived restorativeness to partially mediate the relationship between perceived campus greenness and students' quality of life. Campus restorativeness supports students' psychological restoration and is related to their health measures on campus, concurring with findings of previous studies (19, 20, 32). The present findings indicate perceived restorativeness to be a mechanism that enables students to feel an inner balance, which in turn leads to a positive psychological restoration experience and provides an avenue for positive health measures through multi-sensory perception of campus landscapes qualities.

## Conclusions, Limitations and Suggestions for Further Research

In this paper we determined university students' psychological restoration in outdoor campus green space by identifying the importance of environmental characteristics of PSD and perceived restorativeness. Implementing health promoting and environmental restorative design strategies could provide the potential to improve the mental health of university students and lead to improvements in their quality of life, general health and learning outcomes. We have shown restoration experience to be clearly related to the PSD and perceived restorativeness. However, PSD itself does not appear to have a large impact on restoration experience. The results further illustrate the relevance of perceived restorativeness in relation to restoration experience; more specifically we have shown that restoration experience is enhanced through the impact of PSD on perceived restorativeness–in other words greater effects on restoration experience come through perceived restorativeness that is affected by PSD. While previous studies have suggested PSD to be a potential resource for restorativeness experience and restoration experience, we have demonstrated that although perceived restorativeness is indeed a mechanism to explain this relationship, PSD itself is capable of promoting favorable experiences of restorativeness in a green space and facilitating psychological restorative outcomes.

Our study is the first to measure restoration experience, and its association with PSD and perceived restorativeness in a sample of university students who are facing stress related mental disorders and in real need for frequent restoration experience. It highlights the impact of perceptions of campus green space qualities for students' mental health support in their everyday context. Although we examined restoration experience and its association with PSD and perceived restorativeness both directly and indirectly, we have not examined precisely which perceived dimensions of PSD or perceived restorativeness influence restoration experience in integration with each other. Building on previous studies which have provided limited awareness around the potential of perceived dimensions of PSD and restorativeness characteristics in green spaces for psychological health and well-being, further research could usefully focus on addressing precisely which dimensions of PSD support perceived restorativeness and restoration experience more than others. In this study we have only assessed these relationships at the construct level. Also, this study provided satisfactory levels of internal consistency of perceived dimensions of PSD. Continued research is needed to examine psychometric properties of the scale.

In addition, some of the plausible explanations as to why PSD can generate perceived restorativeness and in turn provide restoration experience, are not yet fully accounted for by this model. The behavioral mechanisms (i.e., spending time) may affect these relationships and provide more insights about why some effects are stronger or weaker than others. Such an approach may also be useful in considering other aspects of human health and well-being.

Nevertheless, the paper makes a significant contribution to the restoration experience literature by establishing a significant mediation effect of perceived restorativeness in the relationship between PSD and restoration experience. The findings also suggest that other mediating factors could be influential in this relationship, which is important in two main respects. First, we suggest the relevance of a mechanistic effect of emotional regulation, as proposed by Johnsen (36), whereby people spend time in a particular setting because it makes them happy or reduces negative emotions. This has obvious ramifications for the role of green spaces in maintaining good mental health, as well as their evident role in the move away from medical and public health models which focus on treatment to a situation that promotes independence and well-being through preventative approaches—and contexts–to health care.

Second, the mediation effects of perceived restorativeness examined here point to the relevance of green spaces in providing psycho-social outcomes such as social cohesion and reduced isolation, which to date have not been systematically captured, or statistically validated. The work described here paves the way for further studies that examine the wider psycho-social value of green spaces through the application of mediation effects and personal sensory dimensions.

## Data Availability Statement

The raw data cannot be made available at this time as it forms part of an on-going study. Following completion of this study the raw data supporting the conclusions of the article can be made available. Any requests should be made to the first author.

## Ethics Statement

Ethical review and approval was not required for the study on human participants in accordance with the local legislation and institutional requirements. Written informed consent for participation was not required for this study in accordance with the national legislation and the institutional requirements.

## Author Contributions

The original research was undertaken by FM as part of her Ph.D. Dissertation, which was supervised by HL. Subsequently PC contributed to the conceptualization, design, presentation and intellectual contribution of the research to current public health debates. MV contributed through provision of detailed presentational comments on the operational and analytical framework, together with expert advice on the statistical analysis presented in the article. All authors contributed to the article and approved the submitted version.

## Conflict of Interest

The authors declare that the research was conducted in the absence of any commercial or financial relationships that could be construed as a potential conflict of interest.
